# Effectiveness of eHealth-Based Psychological Interventions for Depression Treatment in Patients With Type 1 or Type 2 Diabetes Mellitus: A Systematic Review

**DOI:** 10.3389/fpsyg.2021.746217

**Published:** 2022-01-31

**Authors:** Esperanza Varela-Moreno, Mónica Carreira Soler, José Guzmán-Parra, Francisco Jódar-Sánchez, Fermín Mayoral-Cleries, María Teresa Anarte-Ortíz

**Affiliations:** ^1^Departamento de Personalidad, Evaluación y Tratamiento Psicológico, Facultad de Psicología, Universidad de Málaga, Málaga, Spain; ^2^Unidad de Gestión Clínica en Salud Mental, Hospital Regional Universitario de Málaga, Málaga, Spain; ^3^Instituto de Investigación Biomédica de Málaga (IBIMA), Málaga, Spain; ^4^Departamento de Economía Aplicada, Facultad de Ciencias Económicas, y Empresariales Universidad de Málaga, Málaga, Spain

**Keywords:** depression, diabetes mellitus, glycemic control, online, eHealth, telemedicine, psychological treatment, systematic review

## Abstract

**Background:**

Comorbidity between diabetes mellitus and depression is highly prevalent. The risk of depression in a person with diabetes is approximately twice that of a person without this disease. Depression has a major impact on patient well-being and control of diabetes. However, despite the availability of effective and specific therapeutic interventions for the treatment of depression in people with diabetes, 50% of patients do not receive psychological treatment due to insufficient and difficult accessibility to psychological therapies in health systems. The use of information and communication technologies (ICTs) has therefore been proposed as a useful tool for the delivery of psychological interventions, but it continues to be a field in which scientific evidence is recent and controversial. This systematic review aims to update the available information on the efficacy of psychological interventions delivered through ICTs to improve depressive symptomatology in patients with diabetes.

**Methods:**

A systematic review of the literature was performed following the PRISMA guidelines and using MEDLINE, Embase, PubMed, Web of Science, PsycINFO, Scopus, and Cochrane Library databases to search for randomized clinical trials of eHealth treatments for patients with diabetes and comorbid depression from 1995 through 2020. In addition, studies related to follow-up appointments were identified. Inclusion criteria were as follows: (a) randomized clinical trials (RCTs); (b) patients with type 1 and type 2 diabetes; (c) adult population over 18 years of age; (d) presence of depressive symptomatology assessed with standardized instruments; (e) treatments for depression based on established psychotherapeutic techniques and principles; (f) delivered through eHealth technologies. We did not limit severity of depressive symptomatology, delivery setting or comparison group (treatment as usual or other treatment). Two coauthors independently reviewed the publications identified for inclusion and extracted data from the included studies. A third reviewer was involved to discuss discrepancies found. The PEDro scale was used to assess the quality of the RCTs. No meta-analysis of the results was performed. The protocol used for this review is available in PROSPERO (Reg; CRD42020180405).

**Results:**

The initial search identified 427 relevant scientific publications. After removing duplicates and ineligible citations, a total of 201 articles were analyzed in full text. Ten articles met the criteria of this review and were included, obtaining very good scientific quality after evaluation with the PEDro scale. The main results show that the eHealth psychological intervention for depression in patients with diabetes showed beneficial effects both at the end of treatment and in the short (3 months) and long term (6 and 12 months) for the improvement of depressive symptomatology. The methodology used (type of diabetes, eHealth technology used, recruitment context, implementation and follow-up) was very heterogeneous. However, all studies were based on cognitive-behavioral tools and used standardized assessment instruments to evaluate depressive symptomatology or diagnosis of MDD. Glycemic control was assessed by glycosylated hemoglobin, but no benefits were found in improving glycemic control. Only four studies included psychoeducational content on diabetes and depression, but none used tools to improve or enhance adherence to medical prescriptions or diabetes self-care.

**Conclusions:**

ICT-based psychological interventions for the treatment of depression in people with diabetes appear to be effective in reducing depressive symptomatology but do not appear to provide significant results with regard to glycemic control. Nonetheless, the scientific evidence reported to date is still very limited and the methodology very diverse. In addition, no studies have implemented these systems in routine clinical practice, and no studies are available on the economic analysis of these interventions. Future research should focus on studying and including new tools to ensure improvements in diabetes outcomes and not only on psychological well-being in order to advance knowledge about these treatments. Economic evaluations should also be undertaken to analyze whether these treatment programs implemented using eHealth technologies are cost-effective.

## Introduction

Depression is a major global public health problem both because of its high prevalence, with an estimated 300 million people of all ages affected (Friedrich, 2017), and because it carries the highest burden of disability among all mental disorders (Üstün et al., 2001). It is also responsible for the largest proportion of the burden of comorbidity and morbidity attributable to non-fatal health outcomes (Moussavi et al., 2007). The comorbidity between depression and chronic disease, especially diabetes has been widely reported in the literature (Khaledi et al., 2019). The risk of depression in a person with diabetes is approximately double that of a person without this disease (Anderson et al., 2001; Semenkovich et al., 2015), generating a very negative impact on emotional well-being, quality of life, and control of the disease, resulting in poorer diabetes outcomes (Egede and Ellis, 2010). A recent review (Khaledi et al., 2019) found that one in four adults with diabetes had depression and concluded that, given the high prevalence of depressive disorders in patients with diabetes, screening for comorbid depression and its prevalent risk factors in this population is recommended.

Scientific evidence on treatment for depression shows that depression can be successfully treated with a variety of psychological and pharmacological interventions, often implemented through collaborative and stepped care approaches (Cuijpers et al., 2008; Petrak and Herpertz, 2009; Baumeister, 2012). However, despite evidence recommending the combination of both treatments, pharmacological treatments remain the most common option in routine clinical practice. Regarding psychological interventions for depression, those that implement behavioral therapy and cognitive techniques have the highest efficacy and scientific evidence (Hofmann et al., 2012). On the other hand, treatment with antidepressant drugs is shown to be effective, but they differ substantially with respect to short- and long-term side effects (Hackett et al., 2008; Rayner et al., 2010; Moncrieff, 2011; Baumeister, 2012). For this reason, the National Institute for Health and Clinical Evidence (NICE) guideline on depression recommends psychological interventions as first-line treatments for treating depressive symptoms (National Institute for Clinical Excellence, 2009; Baumeister, 2012).

The effective management of diabetes requires a complicated and demanding treatment regimen where the patient must take an active role in his or her self-care, involving a high degree of responsibility for his or her disease and constant decision making about treatment. In addition, each type of diabetes has certain distinct clinical and treatment characteristics. Daily life with diabetes differentiates this disease from other chronic conditions and can generate routines associated with high levels of stress, which can lead to the appearance of depressive symptoms and the need for treatment. The appearance of such depressive symptomatology can also affect glycemic control; however, the data reported to date suggest a mainly indirect effect of depression on glycemic control due to poor self-care behaviors (Snoek et al., 2015). Therefore, treatment of depression in people with diabetes should be oriented toward improving both psychological and medical outcomes, according to the recommendations of the American Diabetes Association (Americam Diabetes Association, 2021).

Psychological interventions aimed at the treatment of depression in patients with diabetes are well-documented as being effective in treating depressive symptoms (Van der Feltz-Cornelis et al., 2010; Markowitz et al., 2011; Petrak et al., 2015). In contrast, studies on pharmacological treatment for depression in people with diabetes report inconclusive results (Baumeister, 2012; Baumeister et al., 2012; Petrak et al., 2015) and sometimes negative results (Lustman et al., 1997). However, reviews on the efficacy of both interventions on improving glycemic control obtained unsatisfactory results (Van der Feltz-Cornelis et al., 2010; Markowitz et al., 2011; Petrak et al., 2015). It is essential to take these data into account for the development of future research and interventions.

Despite the availability of effective therapeutic interventions for the treatment of depressive symptomatology, not all patients can be treated with the resources available. It has been found that 50% of patients are not being treated (Egede and Ellis, 2010), and pharmacological treatment remains the treatment of choice, due to the high cost of face-to-face delivery of these treatments. As a result, healthcare professionals are demanding alternatives for their patients. Recently, in response to the need to improve this situation, non-face-to-face models of alternative psychological interventions have been proposed for implementation in medical care using new information and communication technologies (ICTs), known as eHealth. However, although this is a rapidly advancing field, the scientific evidence is not yet abundant.

Effective online interventions for depression have been designed for the general population (Andrews et al., 2010; Montero-Marín et al., 2016; Karyotaki et al., 2021). However, although eHealth programs to address depressive symptoms in the population with diabetes appear to show improvement in depressive symptomatology and diabetes related distress (Franco et al., 2018), they are scarce and methodologically diverse, offering no data on which aspects are the most effective. Accordingly, this review aims to examine the information published to date on the efficacy of psychological interventions delivered through eHealth to improve depressive symptoms in patients with Type 1 diabetes (T1DM) or Type 2 Diabetes (T2DM) and to analyze the characteristics of each, in order to contribute empirical evidence useful to professionals in their decision-making when developing, designing, or selecting future ICT-based interventions for depression in people with diabetes.

## Objective

The aim of this study was to conduct a systematic review of the effectiveness of eHealth programs designed to reduce depressive symptomatology in people with type 1 diabetes mellitus (T1DM) and type 2 diabetes mellitus (T2DM) compared to control groups (treatment as usual [TAU] or other modalities). To do this, the main outcome evaluated was the change in depressive symptoms assessed by validated psychometric instruments that evaluate depressive symptomatology after the treatment and in the follow up. Secondary objectives were to analyze the effectiveness of the treatments on glycemic control through glycosylated hemoglobin (HbA1c) or other measures of diabetes monitoring.

## Methods

This systematic review was carried out according to the recommendations of the PRISMA statement (Urrútia and Bonfilll, 2013), and the protocol followed to develop this systematic review is available in PROSPERO (Reg: CRD42020180405).

### Inclusion and Exclusion Criteria

The following inclusion criteria were considered for this review: (a) randomized clinical trials (RCTs); (b) patients with a diagnosis of T1DM and T2DM according to ADA criteria (2021); (c) presence of depressive symptomatology or Major Depressive Disorder (MDD) assessed with standardized instruments; (d) adult population over 18 years of age; (e) psychological treatment programs for depression based on established psychotherapeutic techniques and principles; (f) eHealth-based psychological intervention (mobile, web, etc.). There were no limits to the scope of the intervention or to the severity of depressive symptomatology. The inclusion criteria for the control group were as follows: (a) non-exposed control group: TAU or waiting list; (b) comparisons with others equivalent treatments (e.g., face-to-face treatments).

Excluded from this review were all published uncontrolled studies or any research that did not provide results on the effectiveness of these programs (e.g., protocols). We also excluded studies focusing on other types of diabetes (e.g., gestational diabetes), populations under 18 years of age, those aimed at other chronic diseases or psychopathological disorders, studies that did not implement eHealth-based depression treatment programs, studies not aimed at the treatment of depression (e.g., assessment studies, self-help, or psychoeducational treatments) and those that did not use validated assessment instruments.

### Information Sources and Search Strategy

The following databases were used in our search strategy: MEDLINE, Embase, PubMed, Web of Science, PsycINFO, Scopus, and Cochrane Library. Hand searches of reference lists of studies and searches of Internet resources were also performed (e.g., Google Scholar). Electronic searches were performed using various combinations of search terms such as diabetes; depression; depressive disorder; affective symptoms; internet; computer; online therapy; telehealth; telecare; web-based; e-health intervention; blood glucose; glycosylated hemoglobin; glycemic load. The language was not limited, and the years of publication were stipulated to be between 1995 and 2020 (decision based on the recognition that the Internet became a major source in 1995 with the launch of Windows 95). The last search was conducted on December 15, 2020. For example, using PubMed, the specific search strategy was as follows: (((diabetes[Title/Abstract]) AND (depression[Title/Abstract] OR “depressive disorder”[Mesh] OR “affective symptoms”[Mesh])) AND (internet[Title/Abstract] OR computer[Title/Abstract] OR “online therapy”[Mesh] OR “telehealth”[Mesh] OR “telecare”[Mesh] OR “web-based”[Mesh] OR “e-health intervention”[Mesh])) AND (blood glucose[Title/Abstract] OR glycosylated hemoglobin[Title/Abstract] OR “glycemic load”[Mesh].

### Study Selection and Data Extraction Process

**Figure 1:**
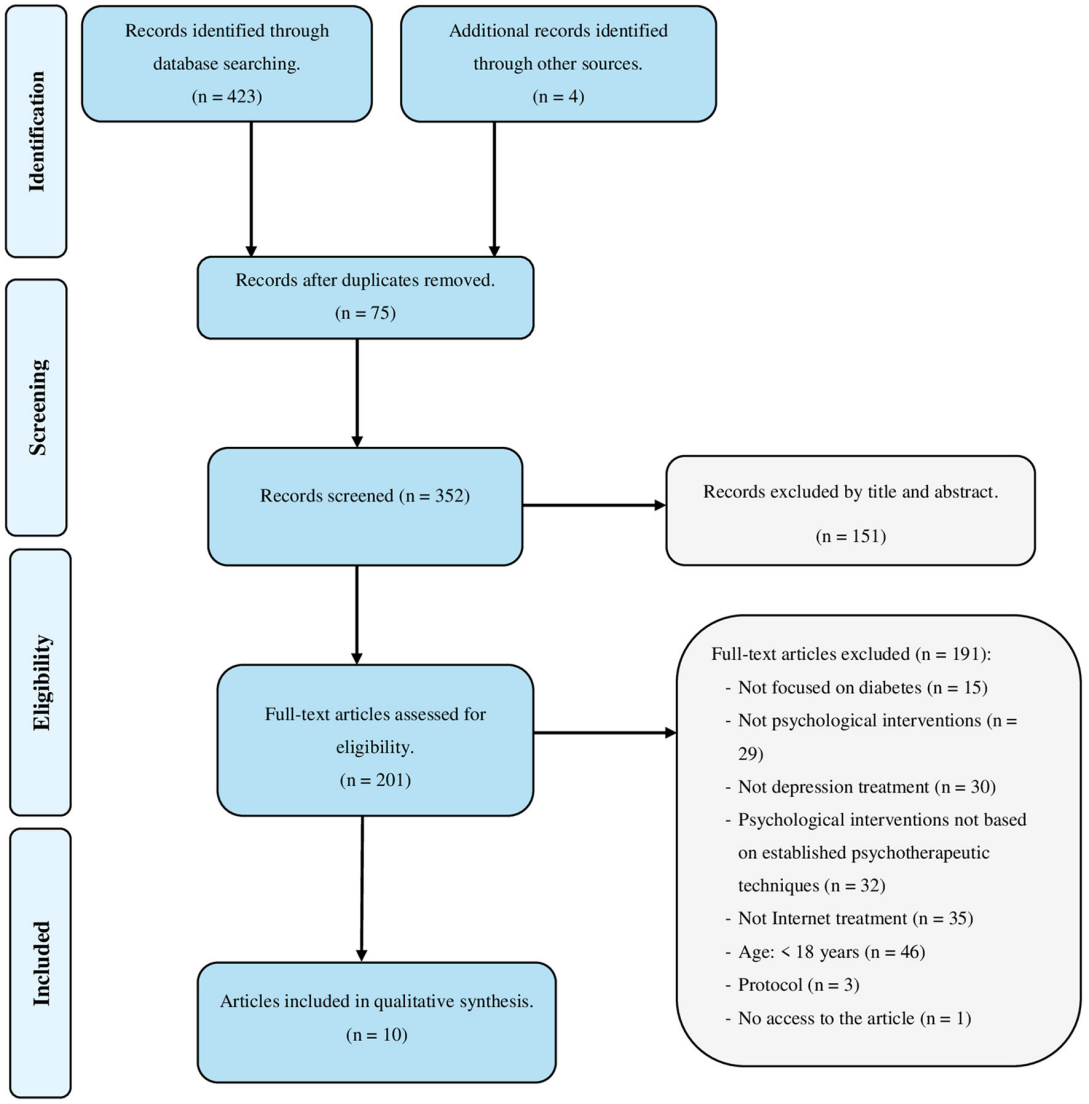
Flow diagram based on PRISMA guidelines.

The studies were selected through a two-stage process. First, two independent reviewers (EV and MC) extracted the data from the different databases and imported them into an application for the management of bibliographic references (Zotero), removing duplicate citations. After obtaining the total number of records or unique citations screened, both reviewers independently examined the titles and abstracts of all the studies generated by the electronic searches. Second, they checked that the inclusion and exclusion criteria were met. Thus, if the abstract met the inclusion criteria, following the protocol for article selection, the full texts were obtained. A third reviewer intervened (MTA), after receiving both reviews, in order to resolve the discrepancies found, and ten articles were finally included for qualitative analysis (see Figure 1).

### Data Analysis

Due to the paucity of studies conducted on eHealth treatments for depression in people with diabetes, the diverse methodology and the insufficient data reported on the effectiveness of the programs provided by the different RCTs, we decided not to conduct a meta-analysis of the results found. Consequently, we were unable to include a quantitative analysis of the results; instead, we conducted a systematic review following the recommendations of the PRISMA guidelines (Moher et al., 2009).

### Evaluation of Studies Quality

The PEDro scale (de Morton, 2009) was used to assess the quality of the clinical trials by means of 11 items rated 0–1, depending on whether the study does not meet the evaluated criterion or meets it, respectively. It should be noted that the first item is not considered for the final calculation, and thus the maximum score is 10 points. Those studies with a total score equal to or higher than 6 were considered *high* quality, those with a total score of 4 or 5 were classified as *moderate* quality, and those with a total score of <4 were considered *low* quality (Maher et al., 2003; de Morton, 2009). For the evaluation of the studies based on these criteria, two independent reviewers (EV, MC) performed the analysis by verifying compliance with the criteria. Any discrepancies found were resolved by a third reviewer (MTA).

## Results

### Search Results

The electronic search yielded 423 potentially relevant articles. Four additional articles were identified that were not found in the databases but obtained after manual searches of reference lists of studies and searches of Internet resources. After removal of duplicates (*n* = 75) and ineligible studies (*n* = 151), a total of 201 articles were retained for full-text review. Fifteen articles were eliminated because they did not focus on diabetes; 29 articles did not evaluate the effectiveness of a psychological intervention; 30 articles did not address the treatment of depression; 32 were not based on established psychotherapeutic techniques; 35 were not delivered using eHealth tools; 46 were not aimed at an adult population over 18 years of age; three articles addressed the protocol and did not report clinical trial data; one article was excluded because it was not possible to access it. Finally, 10 articles were selected for full-text evaluation. The flow diagram is presented in Figure 1.

### Quality of the Studies

Regarding the quality of the included articles, all were of *moderate-high* quality on the PEDro scale. Nine of these articles were considered high quality, scoring above six points on the PEDro scale: Nobis et al. (2015), Clarke et al. (2019), Naik et al. (2019), and Baldwin et al. (2020) scored 9/10. Ebert et al. (2017) achieved a score of 8/10, and in the articles by Piette et al. (2011), Van Bastelaar et al. (2011, 2012), and Newby et al. (2017) the score was 7/10. Finally, the study by Egede et al. (2018) scored 5/10. The evaluation of the studies using the PEDro scale therefore shows values between 5/10 and 9/10. Thus, nine of the articles scored higher than 6/10, and were considered to be of high quality. Only one article scored 5/10, indicating moderate quality. No study was rated as low quality. Consequently, we can conclude that, according to this assessment, the articles included in this review are of high and moderate quality, with the majority being considered high quality (except one). With respect to the analysis of each of the items of the PEDro scale, we note that item 1 (inclusion criteria were specified) and item 2 (subjects were randomly assigned to the groups) were satisfied by all the articles analyzed (Piette et al., 2011; Van Bastelaar et al., 2011, 2012; Nobis et al., 2015; Ebert et al., 2017; Newby et al., 2017; Egede et al., 2018; Clarke et al., 2019; Naik et al., 2019; Baldwin et al., 2020). Item 3 (allocation was concealed) was satisfied by all but three studies (Van Bastelaar et al., 2011, 2012; Egede et al., 2018). Item 4 (the groups were similar at baseline in relation to the most important prognostic indicators) was satisfied by all the articles. Item 5 (all subjects were blinded) was not met in three of the articles (Piette et al., 2011; Van Bastelaar et al., 2011, 2012). Item 6 (all therapists administering treatment were blinded) was only met in six of the articles analyzed (Van Bastelaar et al., 2011, 2012; Nobis et al., 2015; Ebert et al., 2017; Clarke et al., 2019; Baldwin et al., 2020). Item 7 (all evaluators who measured at least one key outcome were blinded) was met in seven of the ten articles (Van Bastelaar et al., 2011, 2012; Nobis et al., 2015; Ebert et al., 2017; Clarke et al., 2019; Naik et al., 2019; Baldwin et al., 2020). Item 8 (measures of at least one of the key outcomes were obtained from more than 85% of the subjects initially assigned to the groups) was only satisfied in three of the articles (Piette et al., 2011; Nobis et al., 2015; Egede et al., 2018). Item 9 (results were presented for all subjects who received treatment or were assigned to the control group, or when this was not possible, data for at least one key outcome were analyzed by intention to treat) was satisfied by seven of the articles (Van Bastelaar et al., 2011, 2012; Ebert et al., 2017; Newby et al., 2017; Clarke et al., 2019; Naik et al., 2019; Baldwin et al., 2020). Item 10 (results of between-group statistical comparisons were reported for at least one key outcome) was not satisfied by one of the articles analyzed (Van Bastelaar et al., 2011). Finally, item 11 (study provides point and variability measures for at least one key outcome) was not satisfied by two of the articles analyzed (Van Bastelaar et al., 2011; Egede et al., 2018).

### Characteristics of Included Studies and Participants

#### Design

The ten articles included (Piette et al., 2011; Van Bastelaar et al., 2011, 2012; Nobis et al., 2015; Ebert et al., 2017; Newby et al., 2017; Egede et al., 2018; Clarke et al., 2019; Naik et al., 2019; Baldwin et al., 2020) corresponded to a total of seven studies. For easier reading we allude to the first published article to refer to each study (Piette et al., 2011; Van Bastelaar et al., 2011; Nobis et al., 2015; Newby et al., 2017; Egede et al., 2018; Clarke et al., 2019; Naik et al., 2019). All the studies included in this review were RCTs published in scientific journals. The search did not yield any doctoral theses or conference proceedings.

#### Recruitment Context

With regard to recruitment, two of the studies analyzed used advertisements and healthcare settings to recruit the sample (Newby et al., 2017; Clarke et al., 2019). Two studies exclusively used advertisements as recruitment methods (Van Bastelaar et al., 2011; Nobis et al., 2015), and three used only the healthcare setting as the recruitment method (Piette et al., 2011; Egede et al., 2018; Naik et al., 2019). None used a primary healthcare system for recruitment.

#### Participants

The sample size of the studies was 1960 patients (Piette et al., 2011; Van Bastelaar et al., 2011; Nobis et al., 2015; Newby et al., 2017; Egede et al., 2018; Clarke et al., 2019; Naik et al., 2019). The characteristics of the included studies and the information extracted are summarized in Table 1.

**Table 1:** Characteristics of the included studies according to the type of eHealth application.

**References**	**Country**	**Sample (** ***N*** **)**		**Type of DM**	**Type of app eHealth**	**Age (*M, SD*)**	**Gender (% female sex)**	**Sample recruitment**	**Depression assessment and criteria**	**Glycemic control**	**Other variables**
		**TG (*n*)**	**CG (*n*)**								
Nobis et al. (2015) and Ebert et al. (2017)	Germany	256		T2DM or T1DM	Web-based	51 (12)	63%	Online and offline advertisement	CES-D ≥23 SCID-I	HbA1c	PAID, HADS, AADQ, DSMQ, CSQ-8
		129	127								
Newby et al. (2017)	Australia	90		T2DM or T1DM	Web-based	46.7 (12.6)	64%	Online advertisements and flyers in medical settings	MDD PHQ (5–23)	HbA1c	PAID, K-10, SF-12, GAD-7, PHQ-15, MINI
		41	49								
Clarke et al. (2019) and Baldwin et al. (2020)	Australia	723		T2DM	Web-based	57.7 (10.6)	60.4%	Online advertisements, community organizations, health professionals	PHQ <19	HbA1c	WAS, DDS, GAD, SMP-T2D
		368	355								
Van Bastelaar et al. (2011, 2012)	Netherlands	255		T2DM or T1DM	Web-based	50 (12)	61%	Advertisements	CES-D ≥16	HbA1c	PAID
		125	130								
Piette et al. (2011)	United States	291		T2DM	Telephone	56 (10.1)	51.1%	Community-university-and VA healthcare system	BDI ≥14	HbA1c	Blood pressure, physical activity (pedometer), Brief Cope, Perceived Competence Scale, Morisky medication adherence scale y SF-12
		145	146								
Naik et al. (2019)	United States	255		T2DM	Telephone	61.9 (8.3)	10.2%	Health care system (MEDVAMC) and outpatient clinics	PHQ-9 ≥10	HbA1c	
		136	89								
Egede et al. (2018)	United States	90		T2DM	Videocall	63.1 (4.2)	2.2%	Health care system (MEDVAMC) and outpatient clinics	MDD (DSM-IV)	HbA1c	BAI, GDS
		43	47								

*TG, Treatment Group; CG, Control/Comparison Group; M, Mean; SD, Standard Deviation; T1DM or T2DM, Type 1 and 2 Diabetes Mellitus; HbA1c, glycosylated hemoglobin; MDD, Major Depressive Disorder. For measurement acronym's meaning, see the List of nomenclatures section*.

All the studies focused on patients with T2DM, and only three also included patients with T1DM (Van Bastelaar et al., 2011; Nobis et al., 2015; Newby et al., 2017). The mean age of the people included in the studies in this review was 52.2 years. Most studies reported that 44.5% of their sample were women (Piette et al., 2011; Van Bastelaar et al., 2011; Nobis et al., 2015; Newby et al., 2017; Clarke et al., 2019), except two studies that reported that 93.8% of the sample were men (Egede et al., 2018; Naik et al., 2019).

#### Evaluation and Monitoring Instruments

All the studies included standardized measures to assess the severity of depressive symptoms. Two studies addressed treatment of mild-moderate depressive symptoms (Piette et al., 2011; Clarke et al., 2019); two had as inclusion criteria, patients with moderate-high severity depressive symptomatology (Van Bastelaar et al., 2011; Naik et al., 2019), and one focused on high severity depressive symptoms (Nobis et al., 2015). Lastly, two of the studies aimed to evaluate the efficacy of treatment for patients with MDD (Newby et al., 2017; Egede et al., 2018).

The following were used to assess depressive symptomatology: the Patient Health Questionnaire-9 item (PHQ-9) (Newby et al., 2017; Clarke et al., 2019; Naik et al., 2019), the Beck Depression Inventory (BDI) (Piette et al., 2011), and the Center for Epidemiological Studies Depression scale (CES-D) (Van Bastelaar et al., 2011; Nobis et al., 2015). In three of the studies, MDD was diagnosed using either the World Health Organization Composite International Diagnostic Interview (WHO CIDI-auto) (Van Bastelaar et al., 2012) or the diagnostic criteria of the Diagnostic and Statistical Manual of Mental Disorders, 4th ed. (DSM-IV) (Egede et al., 2018) or the Mini International Neuropsychiatric Interview 5.0.0 (Newby et al., 2017). Only one study included, in addition to psychometric assessment instruments, the DMS-IV structured clinical interview (SCID-I) to confirm the diagnosis of depression according to the diagnostic criteria of the DMS-IV. The secondary variables evaluated in each of the studies are shown in Table 1.

Glycemic control was analyzed by HbA1c in all the studies. The secondary variables evaluated in each of the studies are shown in Table 1.

### Intervention Characteristics

#### Psychotherapeutic Tools

All the studies included in this review were based on established cognitive and/or behavioral psychological tools for the treatment of depression. The intervention characteristics are listed in Table 2. In four studies, the intervention involved cognitive behavioral therapy (CBT) (Piette et al., 2011; Van Bastelaar et al., 2011; Newby et al., 2017; Clarke et al., 2019), and three studies used a proprietary package that included both cognitive and behavioral strategies: HOPE (Naik et al., 2019), GET.ON Mood Enhancer Diabetes (Nobis et al., 2015), and Behavior Activation Treatment (Egede et al., 2018).

**Table 2:** Characteristics of the intervention and efficacy results in depression and HbA1c.

**Author and type of app**	**Psychotherapeutic**	**Group comparison**	**Follow-up**	**Diabetes content**	**Efficacy results**
Nobis et al. (2015) and Ebert et al. (2017) web-based	Systematic behavioral activation and problem solving	Treatment as usual + online psychoeducation about depression	6 months	Yes	The TG had significantly lower depressive symptoms than the CG at both post-treatment (*d* = 0.89; *p* < 0.001) and 6-month follow-up (*d* = 0.83; *p* < 0.0001) but there were no significant differences with respect to glycemic control.
Newby et al. (2017) web-based	CBT	Treatment as usual	3 months	No	The TG showed statistically significant improvements on the PHQ-9 both at post-treatment (*g* = 0.78) and at 3-month follow-up vs. the CG. No significant differences were found in self-reported HbA1c levels (g = 0.14).
Clarke et al. (2019) and Baldwin et al. (2020) web-based	CBT	Placebo intervention on healthy lifestyles	6 and 12 months	No	All participants showed improvements in depressive symptomatology assessed by the PHQ-9 at post-treatment, but no statistically significant differences were detected between groups (*p* = 0.74) or in HbA1c levels. Efficacy analyses at follow-up report significant improvements at 6 months (*p* < 0.001) and 12 months (*p* < 0.001) between the TG and CG. HbA1c decreased significantly between baseline in the TG and CG and at 6 months (*p* = 0.01) but not at 12 months (*p* = 0.12) between the two groups.
Van Bastelaar et al. (2011, 2012) web-based	CBT	Waiting list	No follow-up	Yes	Web-based CBT was effective in reducing depressive symptoms by intention-to-treat analysis (*d* = 0.29; *p* < 0.001) but had no beneficial effect on glycemic control (*p* > 0.05) or in patients with Major Depressive Disorders.
Piette et al. (2011) telephone	CBT	Enhanced Usual Care	No follow-up	Yes	The results show statistically significant improvements between groups (*p* < 0.0001) after the intervention in depression assessed by the BDI. However, no significant improvements in HbA1c (*p* = 0.7) were observed between groups.
Naik et al. (2019) telephone	HOPE	Enhanced Usual Care	12 months	Yes	The differences in PHQ-9 between HOPE and GC were statistically significant after intervention (*p* = 0.03) and at 12 months (*p* = 0.03) but were not significant for HbA1c between groups at either post-treatment (*p* = 0.08) or 12 months (*p* = 0.83).
Egede et al. (2018) videocall	BAT	Same-room treatment	No follow-up	No	No statistically significant differences were found between BAT and same-room therapy. No significant differences were obtained in either depression scores or HbA1c after 12 months of follow-up between the two groups.

*TG, Treatment Group; CG, Control/Comparison Group; CBT, Cognitive Behavioral Therapy; HOPE, Healthy Outcomes Through Patient Empowerment; BAT, Behavioral Activation Treatment; HbA1c, glycosylated hemoglobin. Articles sorted by year of publication and type of eHealth application. For measurement acronym's meaning, see the List of nomenclatures section*.

Psychoeducational content pertaining to diabetes and depression was addressed in four of the studies included in this review (Piette et al., 2011; Van Bastelaar et al., 2011; Nobis et al., 2015; Naik et al., 2019), while three studies did not include this aspect (Newby et al., 2017; Egede et al., 2018; Clarke et al., 2019). Naik et al. (2019) also included management of diet-related thoughts, physical activity, and medication management.

#### Comparison Group

In the studies analyzed in this review, the treatment group was compared with different group formats: one study compared their intervention vs. waiting list (Van Bastelaar et al., 2011), three studies used enhanced usual care (Piette et al., 2011; Newby et al., 2017; Naik et al., 2019), one study compared the intervention with a treatment-as-usual group plus online psychoeducation for depression (Nobis et al., 2015), another study with a placebo intervention group on healthy lifestyles (Clarke et al., 2019), and one study compared the same intervention but with different formats: video call vs. same-room (Egede et al., 2018). None of the studies reported the specifics of TAU.

#### Type of eHealth Delivery

The eHealth delivery method most commonly used to implement the intervention was based on web tools (Van Bastelaar et al., 2011; Nobis et al., 2015; Newby et al., 2017; Clarke et al., 2019). A further two studies used the telephone (Piette et al., 2011; Naik et al., 2019), and only one study used a video call format (Egede et al., 2018). The duration of the intervention and participant follow-up was also different in each of the studies analyzed, as can be seen in Table 2.

#### Follow-Up

The results of this review indicate that patient follow-up was very diverse. One study evaluated efficacy in the short term (3 months) (Newby et al., 2017), other studies in the medium and long term (6 and 12 months) (Nobis et al., 2015; Clarke et al., 2019; Naik et al., 2019), and three of them (Piette et al., 2011; Van Bastelaar et al., 2011; Egede et al., 2018) reported no follow-up evaluation data for the variables studied, as shown in Table 2.

### Efficacy of Intervention on Depressive Symptomatology and Glycemic Control

#### Depressive Symptomatology

After reviewing the efficacy results of the various eHealth treatment programs for the improvement of depressive symptomatology and MDD in people with T1DM and T2DM in the studies included in this review, we found that all the studies report improvements in depressive symptoms following treatment (Piette et al., 2011; Van Bastelaar et al., 2011; Nobis et al., 2015; Newby et al., 2017; Egede et al., 2018; Clarke et al., 2019; Naik et al., 2019) using cognitive-behavioral toolkits and different eHealth formats. Regarding follow-up, only four studies reported efficacy analyses (Nobis et al., 2015; Newby et al., 2017; Clarke et al., 2019; Naik et al., 2019), showing that these results were maintained in the short (3 months) (Newby et al., 2017), medium (6 months) (Nobis et al., 2015; Clarke et al., 2019), and long term (12 months) (Clarke et al., 2019; Naik et al., 2019). These data are provided in more detail in Tables 2, 3.

**Table 3:** Results of depression and HbA1c baseline, post-treatment, and follow-up.

**Author and type of app**	**Depression baseline** ***M (SD)***		**Depression post-treatment** ***M (SD)***		**Depression follow-up** ***M (SD)***			**HbA1c baseline** ***M (SD)***		**HbA1c post-treatment** ***M (SD)***		**HbA1c follow-up** ***M (SD)***		
	**TG**	**CG**	**TG**	**CG**	**Time**	**TG**	**CG**	**TG**	**CG**	**TG**	**CG**	**Time**	**TG**	**CG**
Nobis et al. (2015) and Ebert et al. (2017): web-based	32.2 (7.0)	31.5 (7.5)	21.1 (8.8)	28.9 (8.7)	6 months	19.8 (9.6)	26.8 (9.4)	7.6% (1.6%)	7.4% (1.3%)	–	–	6 months	7.6% (1.6%)	7.4% (1.4%)
Newby et al. (2017): web-based	15.9 (5.2)	14.3 (5.2)	7.7 (5.0)	11.7 (5.2)	3 months	11.0 (4.5)	NR	7.9% (1.8%)	7.7% (1.8%)	NR	NR	3 months	NR	NR
Clarke et al. (2019) and Baldwin et al. (2020): web-based	11.3 (4.0)	10.7 (4.1)	8.7 (5.6)	8.2 (5.5)	6 months	8.3 (0.3)	8.4 (0.3)	NR	NR	NR	NR	6 months	7.4% (0.1%)	7.2% (0.1%)
					12 months	8.4 (0.3)	8.0 (0.3)					12 months	7.5% (0.1%)	7.2% (0.1%)
Van Bastelaar et al. (2011, 2012): web-based	29 (7)	28 (7)	NR	NR	No follow-up	–	–	7.4% (1.6%)	7.3% (1.6%)	NR	NR	No follow-up	–	–
Piette et al. (2011): telephone	26.7 (7.7)	26.5 (9.9)	14.2 (10.3)	18.6 (10.7)	No follow-up	–	–	7.5% (1.7%)	7.7% (1.7%)	7.7% (1.8%)	7.7% (1.7%)	No follow-up	–	–
Naik et al. (2019): telephone	15.8 (4.2)	16.2 (4.0)	10.9 (6.1)	12.4 (6.0)	12 months	10.1 (6.9)	12.6 (6.5)	9.2% (1.4%)	9.3% (1.5%)	9.1% (1.7%)	8.7% (1.7%)	12 months	8.7% (1.6%)	8.9% (2.0%)
Egede et al. (2018): videocall	27.8 (9.6)	28.4 (10.2)	NR	NR	No follow-up	–	–	6.9% (1.1%)	7.3% (2.0%)	NR	NR	No follow-up	–	–

*TG, Treatment Group; CG, Control/Comparison Group; M, Mean; SD, Standard Deviation; HbA1c, glycosylated hemoglobin; NR, not reported. Articles sorted by year of publication and type of eHealth application*.

Regarding the method of administration, all programs based on ICT formats (web, telephone, and video call) were shown to be effective in reducing depressive symptomatology after intervention compared to TAU (Piette et al., 2011; Nobis et al., 2015; Newby et al., 2017; Naik et al., 2019), compared to small psychoeducational interventions in addition to TAU (Nobis et al., 2015), or compared to healthy lifestyles (Clarke et al., 2019), waiting list (Van Bastelaar et al., 2011) or face-to-face treatment (Egede et al., 2018) (Table 3). Likewise, all eHealth treatment programs were effective in improving symptomatology that was mild-moderate (Piette et al., 2011; Clarke et al., 2019), moderate-severe (Van Bastelaar et al., 2011; Naik et al., 2019), and severe (Nobis et al., 2015), as well as in patients with diabetes and diagnosis of MDD (Newby et al., 2017; Egede et al., 2018). However, none of the studies compared their treatment regimen in patients of varying depressive severity. None of the studies reviewed compared different types of eHealth delivery nor did they report cost-effectiveness analyses of each type of delivery in order to report results on which type of eHealth delivery might be more cost-effective in routine clinical practice. Only one study (Egede et al., 2018) compared the effectiveness of the same treatment program for depression for people with diabetes using cognitive-behavioral tools implemented through ICTs (video call) versus the traditional face-to-face format, finding no significant differences between these two formats.

It was not possible to report data on the efficacy of these interventions for each type of diabetes, since none of the studies analyzed made comparisons between these two populations (T1DM and T2DM).

#### Glycemic Control

The mean baseline HbA1c levels found were 6.64% mmHg. None of the studies indicated, in their inclusion criteria, specific HbA1c levels for participation in their programs. Regarding the effect of the eHealth intervention on glycemic control, no significant improvements were found in any of the studies reviewed (Tables 2, 3).

## Discussion

The aim of this study was to conduct a systematic review of the current evidence on eHealth programs available for the treatment of depression in people with diabetes, and to discuss the procedures and findings extracted, since this is a current and rapidly growing topic in the field of mental health and chronic diseases. Our main findings indicate that, although psychological intervention programs are effective in reducing depressive symptomatology in patients with diabetes, the evidence reported thus far on their delivery through eHealth formats is scarce despite the high incidence of depression in this population (Anderson et al., 2001) and the difficulty in accessing face-to-face interventions (Lehtinen et al., 1997). We also found a great deal of heterogeneity in the methodology used by the studies analyzed: type of diabetes, severity of depressive symptomatology, recruitment and intervention context, treatment content, type of eHealth delivery, comparison group, and follow-up, which makes it difficult to generalize the results and draw conclusions. However, this finding is consistent with that reported by other reviews (Petrak and Herpertz, 2009; Markowitz et al., 2011) on the implementation of these treatments in the usual format. Nevertheless, their methodological quality was very good, a basic result that is very favorable for scientific quality.

An important consideration is that three of the reviewed studies (Van Bastelaar et al., 2011; Nobis et al., 2015; Newby et al., 2017) included patients with T1DM and T2DM. This is relevant because although both conditions belong to the same endocrine disease, they are categorized by the American Diabetes Association (Americam Diabetes Association, 2021), as different etiopathogenetic categories with distinct characteristics and treatments. In the case of T2DM, the main objectives of medical treatment focus on lifestyle modification and administration of oral antidiabetic drugs (Knowler et al., 2009; Americam Diabetes Association, 2021). In contrast, T1DM involves a complicated treatment regimen that additionally requires daily self-monitoring of blood glucose, insulin administration, carbohydrate counting, management of hyperglycemia and hypoglycemia, etc. (Americam Diabetes Association, 2021). These differences between the two types of diabetes have important implications for the individual (differentiated sources of distress), as well as for the design and content of the treatment programs implemented. We therefore recommend that this aspect be considered in future research when designing intervention programs, as it may influence their effectiveness.

There was higher percentage of women than men was observed, which again demonstrates the higher prevalence rate of women with depressive symptomatology (Carreira et al., 2010; Snoek et al., 2015). It was also noted that the different studies report high percentages of the population with a high educational level (university studies), but no data is analyzed or reported regarding this variable, which could influence the effectiveness of and adherence to web-based treatment programs for depression. Therefore, it would be of interest for future research to take this variable into account and report efficacy data comparing groups with different educational levels, in order to advance our understanding of this type of intervention.

Regarding the analysis of the results that were the main focus of this review, we found that treatment programs for depression in people with diabetes implemented using eHealth technology appear to be effective in reducing depressive symptomatology. Nevertheless, it is not possible to draw conclusions regarding which format of eHealth technology is most effective in treating depression for the following reasons. The first is the paucity of studies (only seven included studies) and variability of the formats used (web, mobile phone and video call). Second, no studies have been found comparing different eHealth formats implementing the same treatment program. Only the study by Egede et al. (2018) compared an eHealth format (video call) with face-to-face interventions, finding no significant differences between the two formats. Third, and although it was not the subject under study in this review, none of the studies reported economic assessments that indicate whether these programs are cost-effective, possibly because they were not performed in routine clinical practice. This would be an important aspect to include in future research.

As a positive feature, we found that the treatment programs had in common the use of cognitive-behavioral tools, which constitute the psychological treatment for depression that has been shown to be the most effective in the scientific literature (Markowitz et al., 2011; Petrak et al., 2015) and clinical guidelines (National Institute for Clinical Excellence, 2009), with good results. None of the studies included pharmacological therapy, and the psychoeducational content varied among the different studies.

Concerning the trial setting and recruitment of participants, we found great diversity in the studies analyzed. Recruitment ranged from the use of advertisements in social networks to healthcare settings based on patient lists or only brochures. However, no studies were identified that recruited the sample from primary care (PC), carrying out the intervention in the manner most similar to how such interventions would be carried out in the health care setting. In PC, the prevalence of depression is very high at around 29% (Roca et al., 2009), has a high comorbidity with chronic diseases such as diabetes (Anderson et al., 2001), and is associated with poorer glycemic control (Egede and Ellis, 2010). Moreover, pharmacotherapy remains the treatment of choice in PC for this population, despite scientific evidence that psychotherapy achieves superior long-term results and lower relapse rates (Cuijpers et al., 2019). However, due to this high prevalence, the economic resources required to meet the psychological treatment needs of this population in PC are not feasible (Bower and Gilbody, 2005). For this reason, innovative cost-effective alternatives using ICTs for the treatment of depression in PC (Whiteside et al., 2014; Castro et al., 2015; Montero-Marín et al., 2016; Rodriguez-Pulido et al., 2020) that minimally involve mental health services are being proposed. Nonetheless, it is not possible to draw conclusions from this review with respect to the intervention settings because, although ICTs were used for the treatment of depression in people with diabetes, none of the studies were carried out directly in the PC environment. These data are important factors to consider for future interventions in order to study the possible barriers to implementation of these treatments in PC health systems.

Stepped care models have also been proposed in PC (Bower and Gilbody, 2005), whereby a large proportion of patients are treated first with low-intensity interventions, with significant clinical benefits (García-Herrera et al., 2011). These interventions involve a simpler and easier approach than formal psychotherapies. The contact with patients is shorter, and methods such as the Internet or mobile telephony can be used. In the case of depression, low-intensity interventions are offered to those patients who present mild or moderate depressive symptomatology. In addition, interventions that require less interaction time with the therapist than face-to-face psychotherapy (guided self-help approach) or even no interaction at all (unguided self-help approach) seem to provide very positive results at low cost (Spek et al., 2007; Tate et al., 2009). In this review, we found that only two of the studies analyzed focused on the treatment of mild-moderate depression (Piette et al., 2011; Clarke et al., 2019), two focused on the treatment of moderate-severe symptoms (Van Bastelaar et al., 2011; Naik et al., 2019), one on severe depressive symptoms (Nobis et al., 2015), and three were directed at treatment for MDD (Van Bastelaar et al., 2012; Newby et al., 2017; Egede et al., 2018). However, the different intervention studies examined by this review report good results following the intervention, regardless of the severity of depressive symptomatology. Accordingly, the findings appear to indicate that treatment programs for depression in people with diabetes implemented through eHealth formats are effective in improving depressive symptomatology regardless of severity. These results are very promising because many more patients could benefit. Nevertheless, further research is needed in this regard. In addition, it will be important to include diagnostic interviews based on DSM-5 criteria and not only psychometric instruments, since clinical guidelines recommend that the assessment of depression should not be based only on a mere symptom count (National Institute for Clinical Excellence, 2009).

These results therefore suggest that this technology is effective for the treatment of depression in people with diabetes and has the benefit of providing greater reach and care to a broader patient population. These are very important findings given the scarcity of mental health resources (Bower and Gilbody, 2005). However, scientific evidence indicates that depression in people with diabetes not only has an adverse effect on the person's well-being but also on the clinical outcomes of the disease (Petrak et al., 2015), so treatment should be geared toward improving both psychological and medical outcomes (Petrak and Herpertz, 2009). Nonetheless, the results of this review indicate that these treatments are not effective for improving control of diabetes. These results are similar to those reported by other reviews (Van der Feltz-Cornelis et al., 2010; Markowitz et al., 2011; Baumeister et al., 2012; Petrak et al., 2015), so it may be necessary to review the treatments used in order to provide comprehensive patient management. Considering these results, we asked ourselves the following question: what do these treatments bring to the diabetes setting and how do they differ from those developed for people with depression without diabetes? This answer is key for future research to advance existing knowledge. The scientific literature reports that depression appears to exert its effect on glycemic control in an indirect manner, through poor adherence and self-care behaviors in diabetes (Snoek et al., 2015). However, none of the studies included interventions addressed this aspect in their programs. Therefore, it may be essential for the treatment of depression in people with diabetes to include tools aimed at improving adherence and diabetes self-management together with cognitive-behavioral strategies for the reduction of depressive symptomatology.

### Practical Implications

The results of the present review provide evidence of the beneficial effect of eHealth cognitive-behavioral psychological interventions compared with usual care on the reduction of depressive symptomatology. The evidence regarding glycemic control was heterogeneous and inconclusive across the studies reviewed. We recommend that future trials and clinical intervention in patients with diabetes and depressive symptoms consider these results and investigate the inclusion in their programs of tools for self-care and adherence to diabetes treatment to improve not only the results for psychological well-being but also for medical outcomes. It is also important to distinguish between the two types of diabetes in order to develop specific content for each group as well as cost-effective implementation and evaluation of these programs in routine clinical practice.

### Study Limitations

This review collected data from a limited number of very heterogeneous studies on patients with diabetes receiving treatment to reduce depressive symptoms using eHealth technologies, which made it difficult to perform a meta-analysis. The review summarizes the evidence regarding treatments for depression in a variety of settings, but none conducted in PC systems. The included trials comprise samples with patients with type 1 and type 2 diabetes, do not differentiate between the two types, use various eHealth formats, and do not include strategies aimed at improving adherence and diabetes self-care in their programs. Finally, the present systematic review only concentrated on ehealth depression treatment in adults with diabetes. Other meaningful indicators, such as distress, anxiety, and quality of life, were not analyzed, which limited the examination of the overall effects of technology-based interventions. In addition, although other terms such as “technologies” or “telemedicine” could have been used, these terms did not meet the objectives set out in this review, as they were more general. Therefore, in order to focus our search on articles that specifically target ehealth-delivered treatments, we chose to limit the terms used in the review.

## Conclusions

eHealth interventions have great potential to impact public health. The rising use of the Internet and mobile devices across the world has made these interventions increasingly common. However, the scientific evidence in this field is very limited and recent. In order to draw conclusions, further studies that integrate these treatments into clinical practice are needed, as well as economic analyses of this type of intervention versus the traditional face-to-face model.

## Data Availability Statement

The original contributions presented in the study are included in the article/supplementary material, further inquiries can be directed to the corresponding author.

## Author Contributions

EV-M and MC performed the independently searching process and organized the databases. MA-O intervened in order to resolve the discrepancies found after the search in the databases. EV-M wrote the first draft of the manuscript and was reviewed by MA-O and MC. All authors contributed to conception, design of the study, revised the manuscript, read, and approved the submitted version.

## Funding

The translation of this article was carried out with funding from the Puente Project (B.4) of the University of Malaga, entitled Efficacy and Cost-Effectiveness of a Web Application to treat Depressive symptoms in adults with type 1 Diabetes: A controlled clinical trial (20-02-2021 /28-02-2022).

## Conflict of Interest

The authors declare that the research was conducted in the absence of any commercial or financial relationships that could be construed as a potential conflict of interest.

## Publisher's Note

All claims expressed in this article are solely those of the authors and do not necessarily represent those of their affiliated organizations, or those of the publisher, the editors and the reviewers. Any product that may be evaluated in this article, or claim that may be made by its manufacturer, is not guaranteed or endorsed by the publisher.

## Abbreviations

AADQ: Acceptance and Action Diabetes Questionnaire
BAI: Beck Anxiety Inventory
BDI: Beck Depression Inventory
CES-D: Center for Epidemiological Studies Depression scale
CSQ-8: Client Satisfaction Questionnaire
DDS: Diabetes Distress Scale
DSM-IV: Diagnostic and Statistical Manual of Mental Disorders, 4th ed.
DSMQ: Diabetes Self-Management Questionnaire
GAD: Generalized Anxiety Disorder
GDS: Global Deterioration Scale de Reisberg
HADS: Hospital Anxiety and Depression Scales
K-10: The Kessler Psychological Distress Scale
MINI: Mini International Neuropsychiatric Interview 5.0.0
PAID: Diabetes Distress are the Problem Areas in Diabetes
PHQ-9: Patient Health Questionnaire-9 item
SF-12: Patient health-related quality of life
SMP-T2D: Self-Management Profile for Type 2 Diabetes
WAS: Work and Social Adjustment Scale
WHO CIDI-auto: World Health Organization Composite International Diagnostic Interview.
